# Benchmark Problems of Hyper-Elasticity Analysis in Evaluation of FEM

**DOI:** 10.3390/ma13040885

**Published:** 2020-02-17

**Authors:** Yang Han, Junfeng Duan, Shoumei Wang

**Affiliations:** 1 College of Civil Engineering and Architecture, Henan University of Technology, Zhengzhou 450001, China; junfengduan@126.com; 2 Solid Mechanics Research Center, Beijing University of Aeronautics and Astronautics, Beijing 100191, China; smwang@buaa.edu.cn

**Keywords:** hyper-elasticity, rubber-like materials, strain energy density function, exact solution, verification example

## Abstract

The paper proposes benchmark problems on exact solutions of hyper-elastic analysis, which can be used to evaluate analysis capabilities of rubber-like materials provided by a finite element program or other approximate solution methods. Special attention was concentrated on analysis and derivation of the exact solutions for the thick-walled rubber cylinders under internal pressure and axial extension, the thick-walled rubber balloons under internal pressure and the rubber cylinders under torsion or tension-torsion. Deformation and stress analysis on the above three cases were conducted to provide equations and methods for data processing. Exact standard solutions of the problems combined with the strain energy function of generalized high-order polynomials are given. Numerical examples and evaluation results of two commercial packages that are in common use (ABAQUS and ANSYS) are presented. Good agreements are found in the comparisons between the present exact standard solutions and the simulation results.

## 1. Introduction

Hyper-elasticity is a highly non-linear material property. Structural analysis of hyper-elastic materials is always combined with large deformation. Hence analytical solutions in this field were rarely found and numerical approximate methods, such as FEM (finite element method), become musts in this field. However validation of the approximate formulation and program implementation has to be done using some benchmark problems before practical application of an approximate method [1,2]. Many commercial FEM codes, such as reference [3,4], have provided extensive analysis options and incorporated many complicated demonstration examples regarding hyper-elasticity. However, users may like to understand the reliabilities of the analysis capabilities and correctness of the answers by typical benchmark problems with exact solutions.

In addition, the key issue of an appropriate strain energy function for a specific material is still uncertain. Many research works have been devoted in evaluation of the existing ones [5,6,7] or creation of new ones [8,9,10,11]. Thirdly, new methods of solution are still under development [12,13,14] and need to be implemented in commercial codes and tested by a benchmark problem before application.

Therefore, this paper tries to propose some typical problems of which exact numerical solutions can be obtained and can serve as tools to evaluate availabilities of hyper-elasticity analysis in FEM packages or other approximate solutions. Some of the simplest deformation modes, such as uniaxial extension, pure shear and biaxial extension, etc., which were traditionally used to do material property calibration, can no longer be used as standard solutions to check numerical methods. This paper tries to present some other problems of which solutions can be found by elementary analysis or given in literature. The strain energy functions of a generalized high-order polynomial will then be combined to give exact solutions and numerical examples. Finally the evaluation results of FEM packages ABAQUS and ANSYS will be reported.

## 2. Constitutive Relation of Hyper-Elastic Materials

The theory of constitutive relation of hyper-elastic materials has been developed extensively and a great amount of literatures can be found. Reference [15,16] indicated that ABAQUS and ANSYS, etc. have implemented many forms of the well-known strain energy functions, or potentials in short, in their material constitutive relation libraries for users to choose from. This paper has no attempt to introduce the achievements in this field but pays attention to conduct numerical calculations of some typical problems. Therefore the stress-strain relation from Rivlin [17] is cited and re-written into a form convenient for later derivations and computer programming.

(1) $$t_{\Delta }=2\left(\lambda_{\Delta }^{2}W_{,1}-\lambda_{\Delta }^{-2}W_{,2}\right)+\bar{t}$$

and

(2) $$t_{\Delta }-t_{\Delta +1}=2(\lambda_{\Delta }^{2}-\lambda_{\Delta +1}^{2})(W_{,1}+\lambda_{\Delta +2}^{2}W_{,2})$$

where

*t*_Δ_—True stress;
Δ = 1→2→3→1—cyclical index indicating the principle directions of strain and stress;
*Λ* = Principle stretch;
*W* = Strain energy density function;
*W*_,1_, *W*_,2_ = Derivatives of strain energy density function to invariants of right Cauchy-Green strain tensor;
$\bar{t}$ = Hydrostatic pressure.

There are many different forms of the strain energy density function. As examples, only the high-order polynomial functions will be considered hereafter:

(3) $$W=\sum_{i+j=1}^{n}C_{ij}f_{ij}(J_{1},J_{2})=F^{T}C=C^{T}F$$

where **C** is a vector containing the material constants:

(4) $$C=\left\{\begin{array}{cccccc} C_{10} & C_{01} & C_{20} & C_{11} & C_{02} & \cdots \end{array}\right\}$$

while **F** is a vector containing the base functions of the polynomial:

(5) $$F=\left\{\begin{array}{cccccc} {\bar{J}}_{1} & {\bar{J}}_{2} & {\bar{J}}_{1}^{2} & {\bar{J}}_{1}{\bar{J}}_{2} & {\bar{J}}_{2}^{2} & \cdots \end{array}\right\}{\bar{J}}_{1}=J_{1}-3{\bar{J}}_{2}=J_{2}-3$$

In which the invariants of the right Cauchy-Green strain tensor are:

(6) $$J_{1}=\lambda_{1}^{2}+\lambda_{2}^{2}+\lambda_{3}^{2}J_{2}=\lambda_{1}^{2}\lambda_{2}^{2}+\lambda_{2}^{2}\lambda_{3}^{2}+\lambda_{1}^{2}\lambda_{3}^{2}$$

Taking derivation of Equation (3) with respect to the strain invariants result in

(7) $$W_{,1}=F_{,1}^{T}CW_{,2}=F_{,2}^{T}C$$

with

(8) $$F_{,1}=\left\{\begin{array}{cccccc} 1 & 0 & 2{\bar{J}}_{1} & {\bar{J}}_{2} & 0 & \cdots \end{array}\right\}F_{,2}=\left\{\begin{array}{cccccc} 0 & 1 & 0 & {\bar{J}}_{1} & 2{\bar{J}}_{2} & \cdots \end{array}\right\}$$

Substituting Equation (3) into Equations (1) and (2) gives:

(9) $$t_{\Delta }=O_{\Delta }^{T}C+\bar{t}=C^{T}O_{\Delta }+\bar{t}\;\;\Delta =1,2,3$$

and

(10) $$t_{\Delta }-t_{\Delta +1}=Q_{\Delta }^{T}C=C^{T}Q_{\Delta }\Delta =1\to 2\to 3\to 1$$

in which

(11) $$\begin{array}{l} O_{\Delta }=2\left(\lambda_{\Delta }^{2}F_{,1}-\lambda_{\Delta }^{-2}F_{,2}\right)\\ Q_{\Delta }=2(\lambda_{\Delta }^{2}-\lambda_{\Delta +1}^{2})(F_{,1}+\lambda_{\Delta +2}^{2}F_{,2})\Delta =1\to 2\to 3\to 1 \end{array}$$

## 3. Thick-Walled Rubber Cylinder

**Figure 1:**
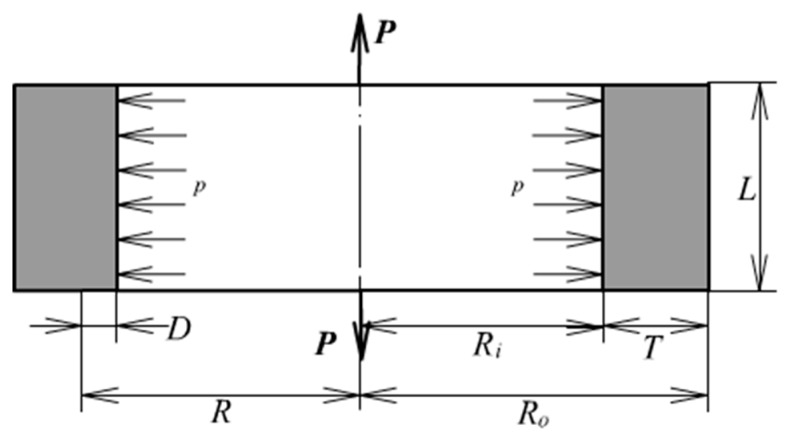
A thick-walled cylinder.

A thick-walled rubber cylinder under internal pressure and axial extension is shown in Figure 1. The dimensions and loads are denoted also. The symbol *P* and *p* denotes axial pulling force and internal pressure. *L*, *R*_0_, *R*_i_ and *T* represent the length, outer radius, inner radius and thickness of the cylinder; *R* is an arbitrary radius; *D* is the distance from an arbitrary point to the inner wall. A fundamental assumption is going to be made: The well-known plane section assumption. Therefore, the deformation mode is quite simple: Uniform axial extension and uniform radial expansion. Besides, under the consideration of fully incompressibility, the deformation is completely defined by only two parameters: Axial extension of the cylinder length and radial expansion of the inner or outer radius.

### 3.1. Deformation Analysis

Apparently, the deformation in the radial direction is non-uniform. Examine the deformation at an arbitrary radius *R*. Let *ξ* be the dimensionless coordinate:

(12) $$\xi =\frac{D}{T}=\frac{R-R_{i}}{R_{o}-R_{i}}=\frac{R-R_{i}}{T}$$

or

(13) $$R=R_{i}+\xi (R_{o}-R_{i})=R_{i}+\xi T$$

Let *r*_i_, *r*_0_ be the inner and outer radius after deformation. Due to full incompressibility, there exists:

(14) $$V=\pi (R_{o}^{2}-R_{i}^{2})L=v=\pi (r_{o}^{2}-r_{i}^{2})l$$

where *l* denotes length of the cylinder after deformation. Hence,

(15) $$r_{i}=\sqrt{r_{o}^{2}-(R_{o}^{2}-R_{i}^{2})\frac{L}{l}}$$

or

(16) $$r_{o}=\sqrt{r_{i}^{2}+(R_{o}^{2}-R_{i}^{2})\frac{L}{l}}$$

An arbitrary radius deforms into:

(17) $$r=r_{i}+\xi (r_{o}-r_{i})=r_{i}+\xi t$$

where *t* is the thickness of the cylinder after deformation.

### 3.2. Strain Analysis

The principle stretch, numbered 1, in axial direction is:

(18) $$\lambda_{1}=\lambda_{a}=\frac{l}{L}$$

The principle stretch in hoop direction, numbered 2, is:

(19) $$\lambda_{2}(\xi )=\lambda_{h}(\xi )=\frac{r}{R}=\frac{r_{i}+\xi (r_{o}-r_{i})}{R_{i}+\xi (R_{o}-R_{i})}=\frac{r_{i}+\xi t}{R_{i}+\xi T}$$

It is worth noting that the hoop strain varies with *ξ*. According to full incompressibility, the principle stretch in the radial direction, numbered 3, is:

(20) $$\lambda_{3}(\xi )=\lambda_{r}(\xi )=\frac{1}{\lambda_{1}\lambda_{2}(\xi )}=\frac{L}{l}\frac{1}{\lambda_{2}(\xi )}$$

which is also a function of *ξ*.

The invariants are:

(21) $$J_{1}={\left(\frac{l}{L}\right)}^{2}+\lambda_{2}^{2}(\xi )+{\left(\frac{L}{l}\right)}^{2}\frac{1}{\lambda_{2}^{2}(\xi )}J_{2}={\left(\frac{L}{l}\right)}^{2}+{\left(\frac{l}{L}\right)}^{2}\lambda_{2}^{2}(\xi )+\frac{1}{\lambda_{2}^{2}(\xi )}$$

### 3.3. Stress Under Pre-Specified Deformation

**Figure 2:**
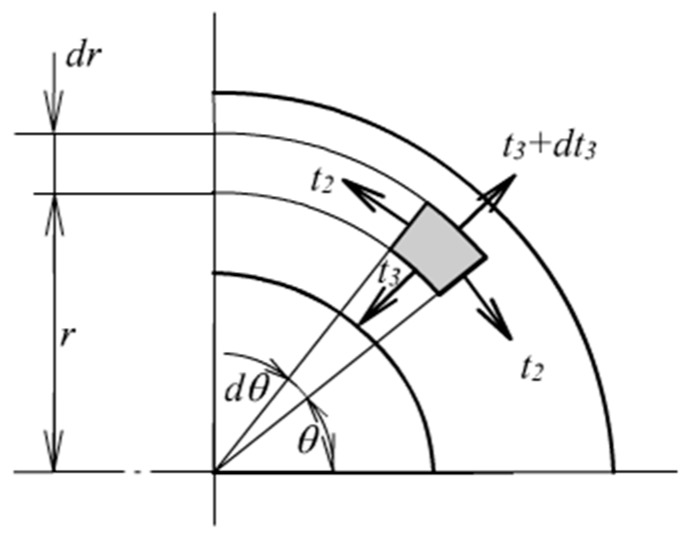
Radial equilibrium after deformation.

The stress can be calculated if *l* and *r*_i_, are known. According to Equations (18)–(21), all of the strain quantities can be obtained. In addition, the stress calculation can start from the radial equilibrium equation. It is well known that in the small deformation regime the equation is [18]:

(22) $$\sigma_{h}-\sigma_{r}=R\frac{d\sigma_{r}}{dR}$$

where *σ* denotes nominal stress in hoop and radial directions. The symbol *R* denotes radius before deformation. In hyper-elasticity analysis, large deformation must be taken into account. The equilibrium relation is then expressed by true stresses and radius after deformation, as shown in Figure 2, where *r* denotes an arbitrary radius after deformation, *t*_2_ and *t*_3_ represent the hoop stress and the radial stress respectively.

The equation becomes:

(23) $$t_{h}-t_{r}=t_{2}-t_{3}=r\frac{dt_{r}}{dr}=r\frac{dt_{3}}{dr}$$

By substitution of Equation (10) with Δ = 2, Δ+1 = 3 into Equation (23), we have:

(24) $$\frac{dt_{3}}{dr}=\frac{1}{t}C^{T}Q_{2}$$

Taking integration in the whole thickness to calculate the internal pressure:

(25) $$p=C^{T}S_{2}$$

where

(26) $$S_{2}=t\int_{0}^{1}\frac{1}{r}Q_{2}d\xi$$

Taking integration in the thickness direction to calculate the radial stress:

(27) $$t_{3}=C^{T}s_{2}-p$$

where

(28) $$s_{2}=t\int_{0}^{\xi }\frac{1}{r}Q_{2}d\xi$$

By substitution of Equation (27) into Equation (10) with Δ = 2, Δ + 1 = 3, we have:

(29) $$t_{2}=t_{3}+C^{T}Q_{2}=C^{T}(s_{2}+Q_{2})-p$$

By substitution of Equation (27) into Equation (10) with Δ = 3, Δ + 1 = 3, we have:

(30) $$t_{1}=t_{3}-C^{T}Q_{3}=C^{T}(s_{2}-Q_{3})-p$$

Finally the axial reaction force is computed by axial equilibrium:

(31) $$P=2\pi \int_{r_{i}}^{r_{o}}t_{1}rdr=2\pi t\int_{0}^{1}t_{1}rd\xi$$

### 3.4. Numerical Example

In order to verify the correctness of the analytical solution, a finite element model of thick-walled rubber cylinder was established. The cylinder, parameters of which are shown in Table 1, deformed uniformly under internal pressure and axial extension.

**Figure 3:**
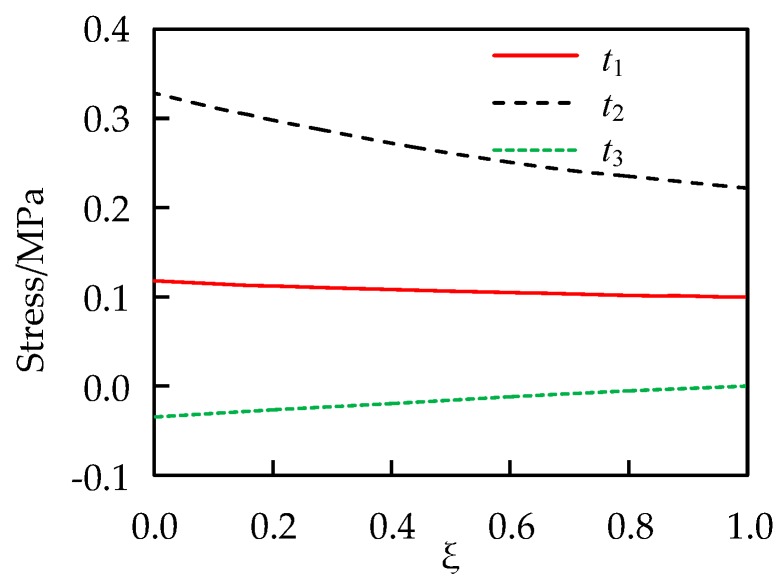
Exact solution of stresses of the cylinder.

The problem is computed by Equations (27), (29)–(31). Very fine intervals are employed when conducting numerical integrations in the radial direction. Figure 3 shows the stress distribution in the radial direction.

**Figure 4:**
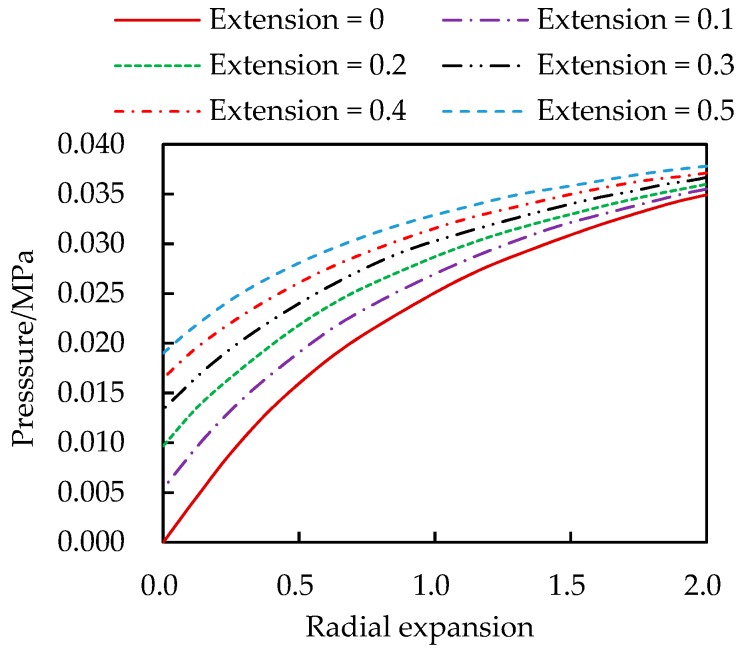
Internal pressure vs. radial expansion and axial extension.

**Figure 5:**
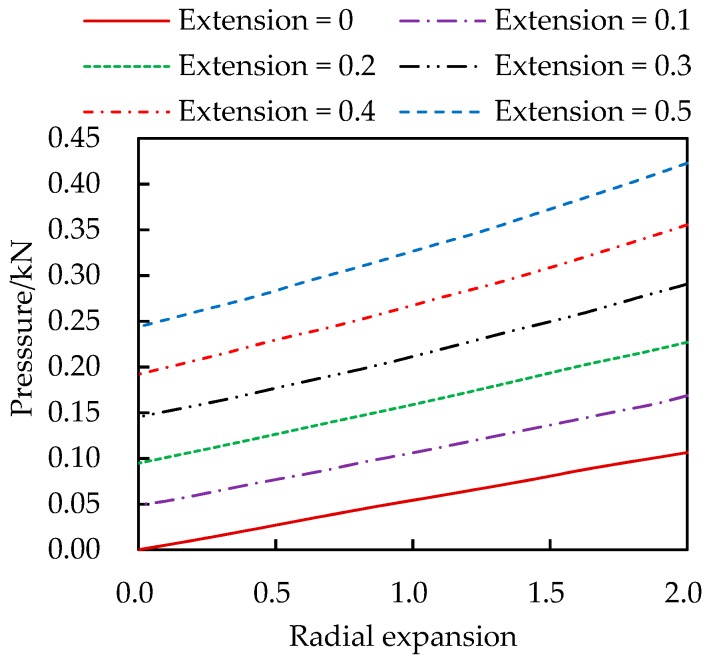
Axial load vs. radial expansion and axial extension.

Figure 4 and Figure 5 show the variations of the internal pressure and axial pulling force with deformations. The radial expansion and axial extension are measured in percentage of the thickness and length, respectively. These curves provide a graphics approach to solve the rubber cylinder problem under action of forces rather than pre-specified displacements. To this end the curves with finer intervals of the axial extension can be drown following the same approach.

**Figure 6:**
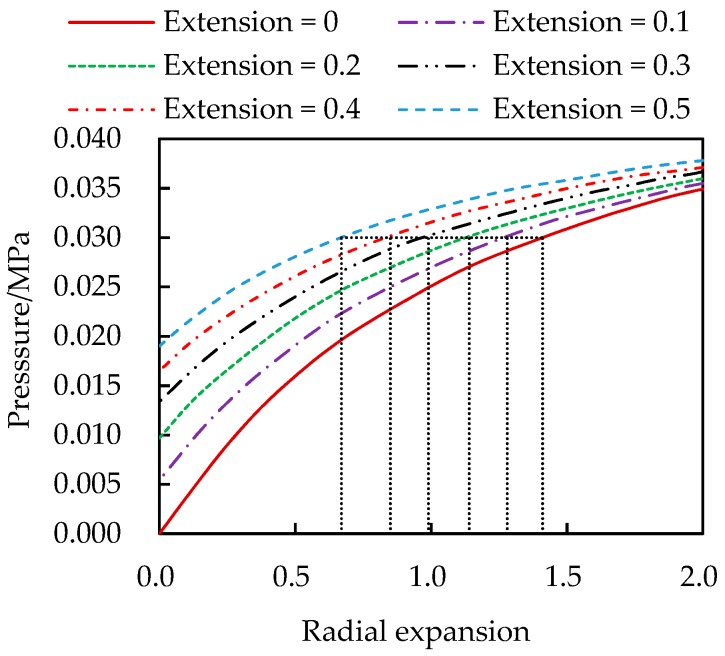
Possible deformation under specific internal pressure.

**Figure 7:**
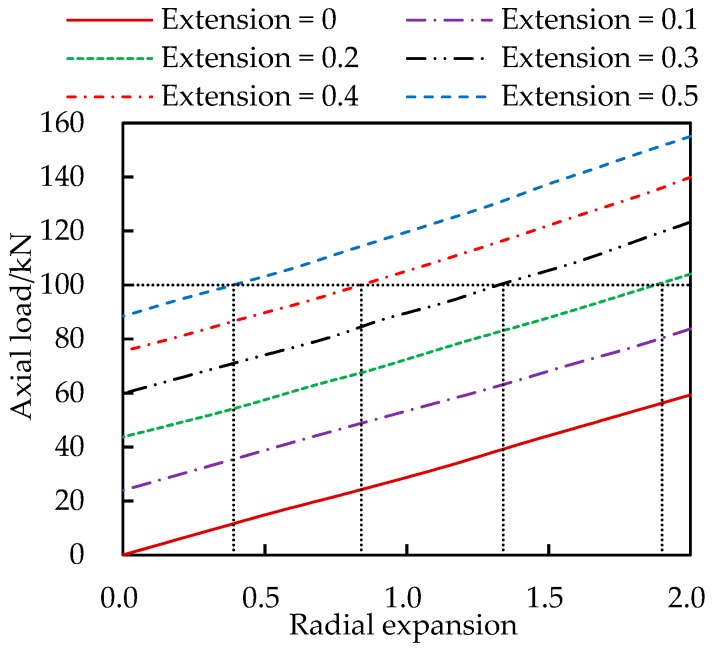
Possible deformation under specific axial force.

As an example, a loading case with axial force *P* = 100 kN and internal pressure *p* = 0.03 MPa is considered. The first step is to draw lines in Figure 6 and Figure 7 to get all possible deformation data under the load *P* or *p*, as shown in Figure 6 and Figure 7. The obtained data are listed in Table 2.

**Figure 8:**
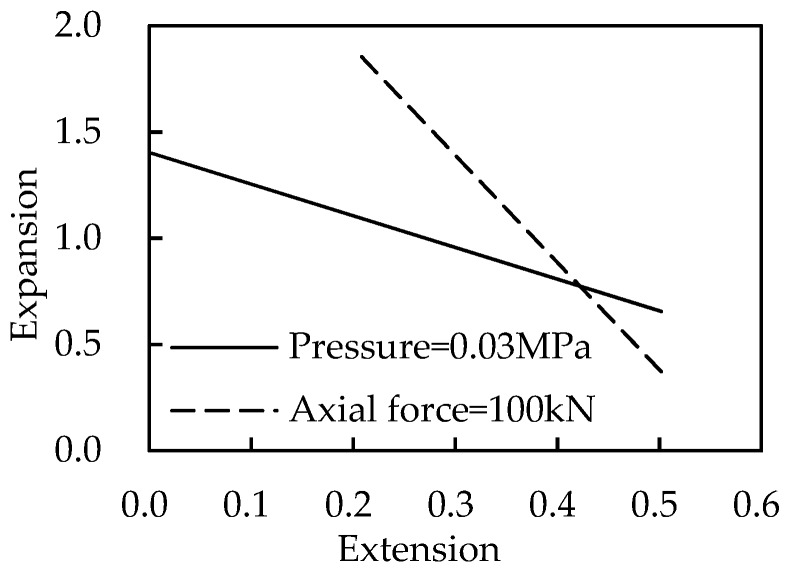
Deformation under specific loads.

In the second step, two lines are drawn based on the data of Table 2, as shown in Figure 8. The answer is located at the intersection point of the two lines, which satisfies the loading *P* = 100 kN and *p* = 0.03 MPa simultaneously.

### 3.5. Evaluation of ABAQUS and ANSYS

**Figure 9:**
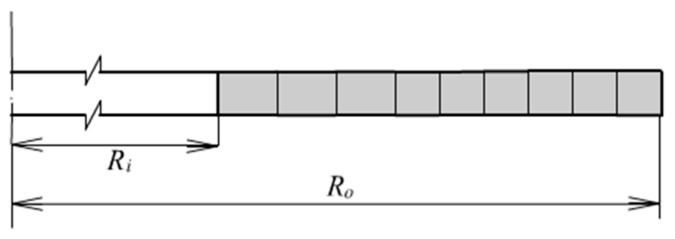
Sketchy of the FEM mesh of the cylinder.

The above results are then used to evaluate FEM codes: ABAQUS and ANSYS. Taking account of uniformity of axial deformation and axisymmetry, the FEM mesh is as shown in Figure 9. In the axial direction, only one element is enough; in the hoop direction, the 2-D axisymmetric elements are adopted. Pre-specified axial displacements are applied on all nodes and pre-specified radial displacements are applied on the two nodes on the inner surface.

Table 3 lists the comparison of the stresses from exact, ABAQUS and ANSYS. The data proved that the two commercial packages provide answers of excellent accuracy.

## 4. Thick-Walled Rubber Balloon

**Figure 10:**
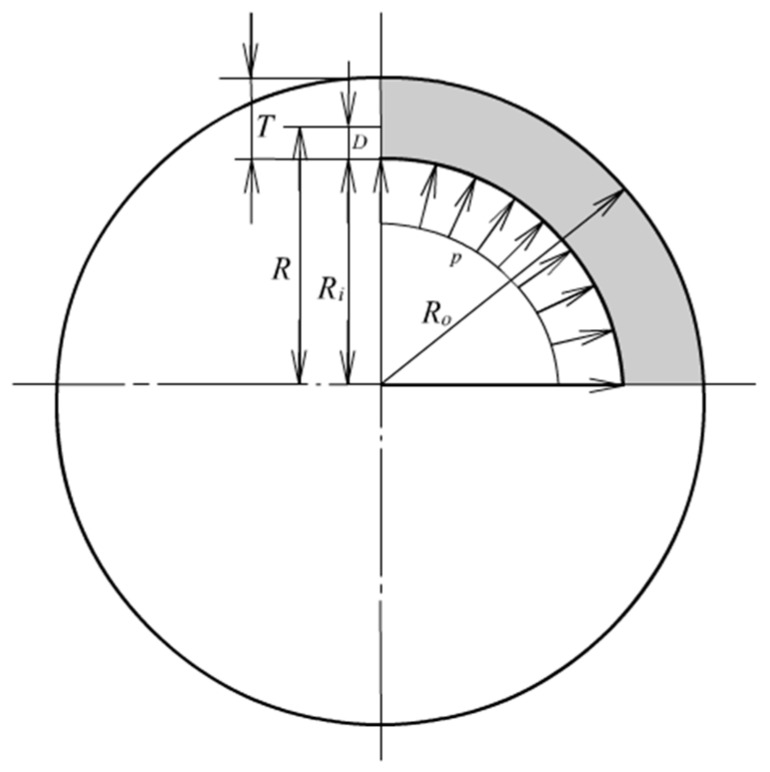
A thick-walled balloon subjected to internal pressure.

A thick-walled rubber balloon under internal pressure is shown in Figure 10. The dimensions and loads are denoted also. The symbols *p* denotes internal pressure. *R*_0_, *R*_i_ and *T* represent the outer radius, inner radius and thickness of the rubber balloon; *R* is an arbitrary radius; *D* is the distance from an arbitrary point to the inner wall. The deformation mode is quite simple: Uniform radial expansion. Besides, under the consideration of full incompressibility, the deformation is completely defined by only one parameter: Radial expansion of the inner or outer radius.

### 4.1. Deformation Analysis

Let *r*_i_, *r*_0_ be the inner and outer radius after deformation, respectively. Due to full incompressibility there exists:

(32) $$V=\frac{4}{3}\pi (R_{o}^{3}-R_{i}^{3})=v=\frac{4}{3}\pi (r_{o}^{3}-r_{i}^{3})$$

Thus, if *r*_0_ is already know:

(33) $$r_{i}=\sqrt[{3}]{r_{o}^{3}-R_{o}^{3}+R_{i}^{3}}$$

Or, if *r*_i_ is already know:

(34) $$r_{o}=\sqrt[{3}]{R_{i}^{3}+R_{o}^{3}-R_{i}^{3}}$$

At an arbitrary radial position, the radius before and after deformation are:

(35) $$R=R_{i}+\xi (R_{o}-R_{i})=r_{i}+\xi T$$

and

(36) $$r=r_{i}+\xi (r_{o}-r_{i})=r_{i}+\xi t$$

Therefore, the hoop stretch in both meridional and circumferential directions is:

(37) $$\lambda (\xi )=\lambda_{1}(\xi )=\lambda_{2}(\xi )=\frac{r}{R}=\frac{r_{i}+\xi (r_{o}-r_{i})}{R_{i}+\xi (R_{o}-R_{i})}=\frac{r_{i}+\xi t}{R_{i}+\xi T}$$

In the radial direction the principle stretch is:

(38) $$\lambda_{3}=\frac{1}{\lambda_{2}\lambda_{3}}=\lambda^{-2}$$

The strain invariants are:

(39) $$J_{1}(\xi )=2\lambda^{2}+\lambda^{-4}J_{2}(\xi )=2\lambda^{-2}+\lambda^{4}$$

### 4.2. Strain Analysis

In view of Equations (37)–(39), Equations (9)–(11) now become:

(40) $$\begin{array}{c} t_{1}=t_{2}=O^{T}C+\bar{t}=C^{T}O+\bar{t}\\ t_{3}=O_{3}^{T}C+\bar{t}=C^{T}O_{3}+\bar{t} \end{array}$$

(41) $$t_{1}-t_{3}=t_{2}-t_{3}=Q^{T}C=C^{T}Q$$

and

(42) $$\begin{array}{c} O=O_{1}=O_{2}=2\left(\lambda^{2}F_{,1}-\lambda^{-2}F_{,2}\right)O_{3}=2\left(\lambda^{-4}F_{,1}-\lambda^{4}F_{,2}\right)\\ Q=2(\lambda^{2}-\lambda^{-4})(F_{,1}+\lambda^{2}F_{,2})=-Q_{3} \end{array}$$

### 4.3. Stress Under Pre-Specified Deformation

Suppose the balloon is under action of a pre-specified expansion of the inner radius: *R*_i_→*r*_i_. All the strain quantities can then be calculated based on Equations (34)–(39).

**Figure 11:**
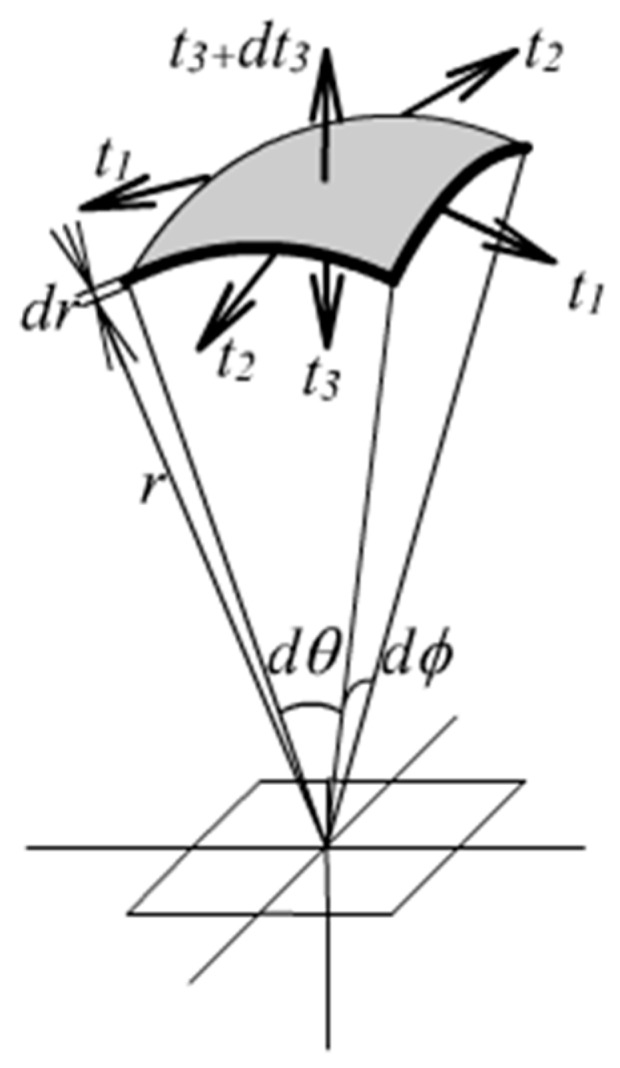
Radial equilibrium of a thick-walled balloon.

Similar to the cylinder problem the stress calculation can start from examining the radial differential Equation. Figure 11 shows an infinitesimal slice cut out from the balloon. Examining the radial equilibrium results in the following Equation:

(43) $$t_{1}-t_{3}=t_{2}-t_{3}=\frac{r}{2}\frac{dt_{3}}{td\xi }$$

Substituting the constitutive Equation (41) into (43), one can obtain:

(44) $$\frac{dt_{3}}{d\xi }=\frac{2t}{r}C^{T}Q$$

By integration along the whole thickness, the internal pressure inducing the pre-specified expansion is:

(45) $$p=C^{T}S$$

In which,

(46) $$S=2t\int_{0}^{1}\frac{1}{r}Qd\xi$$

By integration along a part of the thickness, the true radial stress is obtained.

(47) $$t_{3}=C^{T}s-p$$

In which,

(48) $$s=2t\int_{0}^{\xi }\frac{1}{r}Qd\xi$$

Combining Equation (47) with (41), the hoop or tangential stress is

(49) $$t_{1}=t_{2}=t_{3}+C^{T}Q$$

### 4.4. Numerical Example

**Figure 12:**
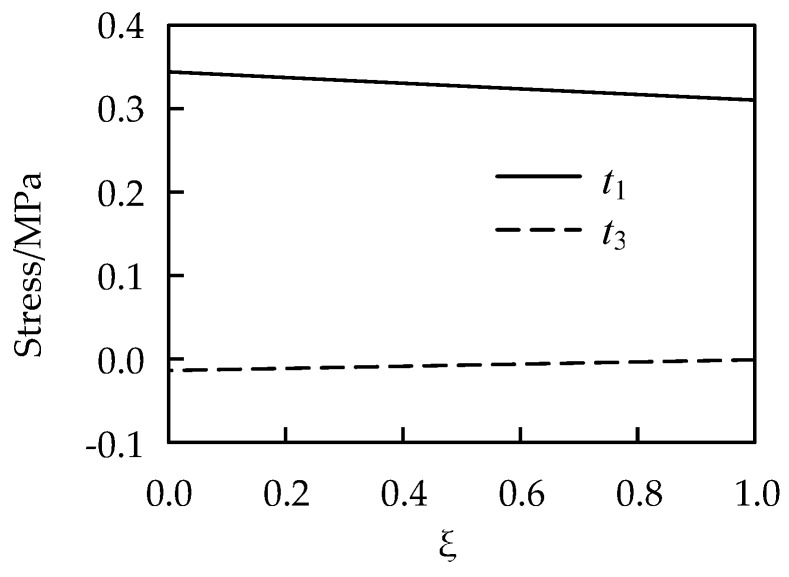
Exact solution of stresses of the balloon.

**Figure 13:**
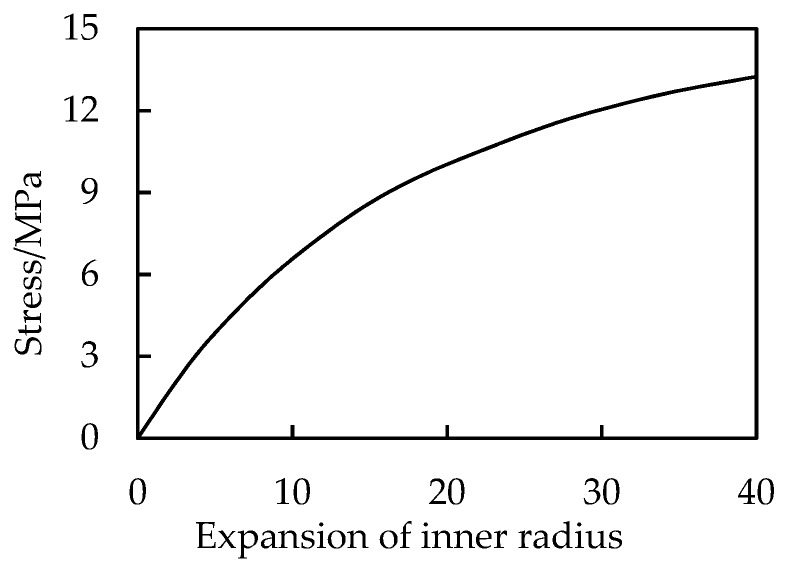
Internal pressure vs. radial expansion.

A thick-walled rubber balloon under internal pressure was established to verify the correctness of the analytical solution. As an example, the rubber balloon with the following parameters (Table 4) were computed:

The problem is computed by Equations (45), (47) and (49). Figure 12 shows the stress distribution in the thickness direction. Figure 13 shows the curve of the internal pressure versus radial expansion. It can also serve as a graphic tool calculating radial expansion of the balloon under action of known internal pressure.

### 4.5. Evaluation of ABAQUS and ANSYS

**Figure 14:**
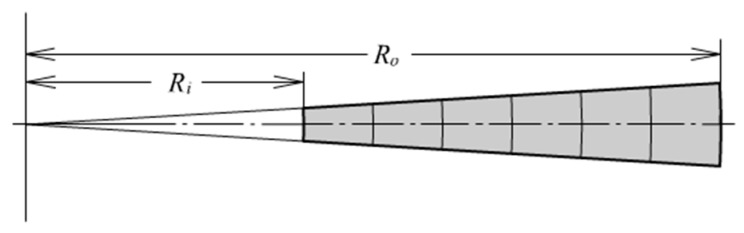
Sketch of the FEM mesh of the balloon.

The example has been calculated by ABAQUS and ANSYS. The adopted mesh is quite similar to the one used in the cylinder problem studied in the last section. But the cross section is now a trapezoid rather than a rectangle, as shown in Figure 14. All the nodes are fixed tangentially. Pre-specified radial displacements are applied on the two nodes on the inner surface.

Comparison of the calculated stresses is made in Table 5. It can be seen that both the software packages possess strong capabilities in hyper-elasticity analysis.

## 5. Simple Torsion and Combined Tension-Torsion of Cylinders

### 5.1. Shear Stress and Torque Under Simple Torsion

**Figure 15:**
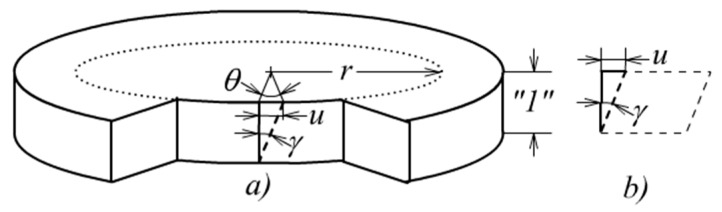
(**a**) Shear deformation of the cylinder and (**b**) Shear deformation of micro element.

When a cylinder, either hollow or solid, is subjected to twist, the main deformation mode is shear. Let us examine a segment of a cylinder with unit length as shown in Figure 15. An arbitrary material point located on a circle of radius *r* will move a distance *u* in the hoop direction, see Figure 15a. Hence a rectangular element on the cylindrical surface will develop the deformation mode of well known “simple shear”, as shown in Figure 15b. The expression of shear stress can then be copied from the relevant references.

(50) $$t_{ha}=2u(W_{,1}+W_{,2})$$

Let the relative twist angle be denoted by *θ* then from Figure 15a:

(51) u=rθ

so

(52) $$t_{ha}=2r\theta (W_{,1}+W_{,2})$$

By integration, the torque is:

(53) $$M=2\pi \int_{r}t_{ha}r^{2}dr=4\pi \theta \int_{r}(W_{,1}+W_{,2})r^{3}dr$$

By substitution of Equations (7) and (8) into (53)

(54) $$M=4\pi \theta C^{T}M$$

with

(55) $$M=\left\{\begin{array}{cccccc} m_{4} & m_{4} & 2m_{6}\theta^{2} & 2m_{6}\theta^{2} & 2m_{6}\theta^{2} & \cdots \end{array}\right\}m_{k}=\frac{1}{k}(R_{o}^{k}-R_{i}^{k})$$

### 5.2. Poynting Effect

It must be pointed out that there is also normal stress acting in the plane of the shear stress due to the so called “Poynting effect”. An elaborate analysis of all the problems can be found in, for instance, Reference [20,21]. The final forms of the solutions are as follows.

Normal force under simple torsion:

(56) $$A=-2\pi \theta^{2}\int_{r}r^{3}(W_{,1}+2W_{,2})dr$$

with

(57) $$J_{1}=J_{2}=3+{\left(r\theta \right)}^{2}$$

Torque and normal force under combined tension-torsion:

(58) $$\begin{array}{l} M=4\pi \theta \int_{r}r^{3}(W_{,1}+\lambda^{-1}W_{,2})dr\\ A=4\pi (\lambda -\lambda^{-2})\int_{r}r(W_{,1}+\lambda^{-1}W_{,2})dr-2\pi \theta^{2}\int_{r}r^{3}(W_{,1}+2\lambda^{-1}W_{,2})dr \end{array}$$

with

(59) $$\begin{array}{l} {\bar{J}}_{1}=\Lambda_{1}+\lambda {\left(r\theta \right)}^{2}{\bar{J}}_{2}=\Lambda_{2}+{\left(r\theta \right)}^{2}\\ \Lambda_{1}=\lambda^{2}+2\lambda^{-1}-3\Lambda_{2}=2\lambda +\lambda^{-2}-3 \end{array}$$

Substitution of Equations (7) and (8) into (56) and (58) yields the following final expressions.

Normal force under simple torsion:

(60) $$\begin{array}{l} A=-2\pi \theta^{2}C^{T}N\\ N=\left\{\begin{array}{cccccc} m_{4} & 2m_{4} & 2m_{6}\theta^{2} & 3m_{6}\theta^{2} & 4m_{6}\theta^{2} & \cdots \end{array}\right\}m_{k}=\frac{1}{k}(R_{o}^{k}-R_{i}^{k}) \end{array}$$

Torque under tension-torsion:

(61) $$\begin{array}{l} M=4\pi \theta C^{T}M\\ M=\{\begin{array}{cccc} m_{4} & \lambda^{-1}m_{4} & 2(\Lambda_{1}m_{4}+m_{6}\theta^{2}\lambda ) & m_{4}(\lambda^{-1}\Lambda_{1}+\Lambda_{2})+2m_{6}\theta^{2} \end{array}\\ \begin{array}{cc} 2\lambda^{-1}(\Lambda_{2}m_{4}+m_{6}\theta^{2}) & \cdots \end{array}\} \end{array}$$

Normal force under tension-torsion:

(62) $$\begin{array}{l} A=4\pi (\lambda -\lambda^{-2})C^{T}N_{a}-2\pi \theta^{2}C^{T}N_{b}\\ N_{a}=\{\begin{array}{cccc} m_{2} & \lambda^{-1}m_{2} & 2(\Lambda_{1}m_{2}+m_{3}\theta^{2}\lambda ) & m_{2}(\lambda^{-1}\Lambda_{1}+\Lambda_{2})+2m_{3}\theta^{2} \end{array}\\ \begin{array}{cc} 2\lambda^{-1}(\Lambda_{2}m_{2}+m_{3}\theta^{2}) & \cdots \end{array}\}\\ N_{b}=\{\begin{array}{cccc} m_{4} & 2\lambda^{-1}m_{4} & 2(\Lambda_{1}m_{4}+\lambda m_{6}\theta^{2}) & (\Lambda_{2}+2\lambda^{-1}\Lambda_{1})m_{4}+3m_{6}\theta^{2} \end{array}\\ \begin{array}{cc} 4\lambda^{-1}(\Lambda_{2}m_{4}+m_{6}\theta^{2}) & \cdots \end{array}\} \end{array}$$

### 5.3. Numerical Example and Evaluation of ABAQUS

Examine a solid or hollow shaft with the following given data (units are in mm, MPa.): Outer radius 10, inner radius 0 or 2, length *l* = 1, material constants are the same with that used in examples of the forgoing sections, relative twist angle = 5°. The problem was calculated by the analytical solution expressed by Equations (54), (60) and ABAQUS. For ABAQUS the asymmetric element CGAX4H was used to model the shaft. The computed shear stress, torque and normal force are listed in Table 6, which exhibit excellent agreement between the two solutions.

The solid cylinder was also computed under a combined tension-torsion with axial stretch equal to 1.5. Results of the torque and normal force are listed in Table 7, which also show good correlation between the analytical solution and answers from ABAQUS.

## 6. Other Forms of the Strain Energy Function

Even though only the polynomial function is discussed in the foregoing sections, the benchmark problems combined with other forms of the strain energy function can be solved in the same manner. First, we should notice that some other functions are nothing but subsets of the polynomial form. In other words, they are incomplete polynomial expressions with some terms missing. This is summarized in Table 8.

By combining the table with Equations (3)–(6), as well as all the related subsequent expressions, the formulation of other strain energy functions can be derived.

## 7. Conclusions

The benchmark problems, thick-walled rubber cylinders under axial extension and/or radial expansion, thick-walled rubber balloons under radial expansion and solid or hollow rubber cylinders under torsion or tension-torsion have been studied. For the strain energy function of generalized polynomial form, the paper gives exact solutions of the problems. Numerical examples and evaluation results of two commercial packages that are in common use are presented. The results were then used to evaluate FEM analysis capabilities of ABAQUS and ANSYS in the field of hyper-elasticity. Good agreements are found in the comparisons between the present exact standard solutions and the simulation results, which can serve as tools to evaluate availabilities of hyper-elasticity analysis in FEM packages or other approximate solutions.

## Figures and Tables

**Table 1:** Example parameters of the cylinder.

Dimensions	Magnitude (mm)	Material Constants [19]	Magnitude (MPa)
Length	200	*C*_10_	450,107 × 10^−1^
Outer radius	20	*C*_01_	258,689 × 10^−1^
Thickness	5	*C*_20_	281,269 × 10^−2^
Length after deformation	500	*C*_11_	905,309 × 10^−3^
Inner radius after deformation	25	*C*_02_	323,452 × 10^−4^

**Table 2:** Possible deformation data under specific loads.

*P* = 100 kN		*p* = 0.03 MPa	
Extension	Expansion	Extension	Expansion
0.20	1.90	0.00	1.40
0.30	1.34	0.10	1.27
0.40	0.84	0.20	1.12
0.50	0.39	0.30	0.97
−	−	0.40	0.82
−	−	0.50	0.67

**Table 3:** Stress comparison of the cylinder (MPa).

*ξ*	Radial			Axial			Hoop		
	Exact	ABAQUS	ANSYS	Exact	ABAQUS	ANSYS	Exact	ABAQUS	ANSYS
0.0	−0.0351	−0.0342	−0.0344	0.1180	0.1181	0.1179	0.3290	0.3277	0.3281
0.1	−0.0304	−0.0304	−0.0302	0.1155	0.1152	0.1147	0.3136	0.3127	0.3115
0.2	−0.0261	−0.0263	−0.0261	0.1132	0.1124	0.1119	0.2995	0.2977	0.2966
0.3	−0.0221	−0.0225	−0.0223	0.1110	0.1099	0.1095	0.2866	0.2842	0.2832
0.4	−0.0183	−0.0188	−0.0186	0.1089	0.1076	0.1073	0.2746	0.2720	0.2711
0.5	−0.0148	−0.0153	−0.0151	0.1069	0.1056	0.1053	0.2636	0.2609	0.2601
0.6	−0.0115	−0.0120	−0.0118	0.1051	0.1038	0.1035	0.2533	0.2508	0.2501
0.7	−0.0083	−0.0088	−0.0086	0.1033	0.1022	0.1019	0.2437	0.2417	0.2410
0.8	−0.0054	−0.0057	−0.0055	0.1015	0.1007	0.1005	0.2348	0.2333	0.2327
0.9	−0.0026	−0.0028	−0.0026	0.0999	0.0994	0.0992	0.2264	0.2256	0.2250
1.0	0.0000	−0.0003	−	0.0983	0.0984	−	0.2185	0.2191	−

Notes: Stresses at the integration points, small deviation from nodes.

**Table 4:** Example parameters of the balloon.

Dimensions	Magnitude (mm)	Material Constants [19]	Magnitude (MPa)
−	−	*C*_10_	450,107 × 10^−1^
Outer radius	100	*C*_01_	258,689 × 10^−1^
Thickness	5	*C*_20_	281,269 × 10^−2^
Inner radius after deformation	135	*C*_11_	905,309 × 10^−3^
−	−	*C*_02_	323,452 × 10^−4^

**Table 5:** Stress comparison of the balloon (MPa).

*ξ*	Radial Stress			Hoop Stress		
	Exact	ABAQUS	ANSYS	Exact	ABAQUS	ANSYS
0.0000	−0.013336	−0.012485	−0.017355	0.343355	0.343900	0.338940
0.1000	−0.011994	−0.011232	−0.011831	0.339950	0.340600	0.339650
0.2000	−0.010672	−0.009941	−0.010231	0.336600	0.337200	0.336520
0.3000	−0.009370	−0.008660	−0.008606	0.333304	0.333800	0.333520
0.4000	−0.008087	−0.007391	−0.007463	0.330060	0.330600	0.330120
0.5000	−0.006824	−0.006132	−0.006435	0.326867	0.327400	0.326690
0.6000	−0.005580	−0.004885	−0.004847	0.323723	0.324200	0.323910
0.7000	−0.004355	−0.003648	−0.003641	0.320629	0.321200	0.320830
0.8000	−0.003148	−0.002421	−0.002437	0.317581	0.318200	0.317830
0.9000	−0.001958	−0.001205	−0.001146	0.314579	0.315300	0.314990
1.0000	−0.000787	−0.000053	−	0.311622	0.312600	−

Notes: Stresses at integration points.

**Table 6:** Shear stress (MPa), torque (kN·mm) and normal force (kN) of simple torsion

*ξ*	Solid Cylinder		Hollow Cylinder	
	Exact	ABAQUS(CGAX4H)	Exact	ABAQUS(CGAX4H)
0.00	0.00000	−8.59370 × 10^−7^	0.02478	0.02478
0.10	0.01238	0.01237	0.03475	0.03475
0.20	0.02478	0.02478	0.04478	0.04477
0.30	0.03725	0.03724	0.05487	0.05486
0.40	0.04981	0.04980	0.06505	0.06505
0.50	0.06250	0.06249	0.07534	0.07533
0.60	0.07534	0.07532	0.08574	0.08573
0.70	0.08836	0.08835	0.09628	0.09627
0.80	0.10161	0.10160	0.10697	0.10700
0.90	0.11510	0.11510	0.11783	0.11780
1.00	0.12887	0.12880	0.12887	0.12880
M	199.72	199.70	199.72	199.4
A	11.76	11.76	11.74	11.66

**Table 7:** Torque (kN·mm) and normal force (kN) of tension-torsion.

Type	Exact	ABAQUS(CGAX4H)
M	186.9	186.9
A	33.08	34.54

**Table 8:** Generalized polynomial functions.

*W*	MR	P2	P3	NH	RP2	RP3	STTS35	STTS37	STTS48
$C_{10}{\bar{J}}_{1}$	√	√	√	√	√	√	√	√	√
$C_{01}{\bar{J}}_{2}$	√	√	√	−	−	−	√	√	√
$C_{20}{\bar{J}}_{1}^{2}$	−	√	√	−	√	√	√	√	√
$C_{11}{\bar{J}}_{1}{\bar{J}}_{2}$	−	√	√	−	−	−	√	√	√
$C_{02}{\bar{J}}_{2}^{2}$	−	√	√	−	−	−	−	√	√
$C_{30}{\bar{J}}_{1}^{3}$	−	−	√	−	−	√	√	√	√
$C_{21}{\bar{J}}_{1}^{2}{\bar{J}}_{2}$	−	−	√	−	−	−	−	√	√
$C_{12}{\bar{J}}_{1}{\bar{J}}_{2}^{2}$	−	−	√	−	−	−	−	−	−
$C_{03}{\bar{J}}_{2}^{3}$	−	−	√	−	−	−	−	−	−
$C_{40}{\bar{J}}_{1}^{4}$	−	−	−	−	−	−	−	−	√

Notes: MR—Mooney-Rivlin; P2, P3—second- and third-order polynomial; RP2, RP3—second- and third-order reduced polynomial; NH—Neo-Hookean; STTS35, STTS37, STTS48—functions proposed by reference [22]; √—the term is present; Blank—the term is ignored.
